# The Metamorphic Nature of the Tau Protein: Dynamic Flexibility Comes at a Cost

**DOI:** 10.3389/fnins.2016.00003

**Published:** 2016-01-21

**Authors:** Jonathan J. Sabbagh, Chad A. Dickey

**Affiliations:** Department of Molecular Medicine, Byrd Alzheimer's Research Institute, University of South FloridaTampa, FL, USA

**Keywords:** tau, microtubules, structure, neurodegenerative diseases, NMR

## Abstract

Accumulation of the microtubule associated protein tau occurs in several neurodegenerative diseases including Alzheimer's disease (AD). The tau protein is intrinsically disordered, giving it unique structural properties that can be dynamically altered by post-translational modifications such as phosphorylation and cleavage. Over the last decade, technological advances in nuclear magnetic resonance (NMR) spectroscopy and structural modeling have permitted more in-depth insights into the nature of tau. These studies have helped elucidate how metamorphism of tau makes it ideally suited for dynamic microtubule regulation, but how it also facilitates tau self-assembly, oligomerization, and neurotoxicity. This review will focus on how the distinct structure of tau governs its function, accumulation, and toxicity as well as how other cellular factors such as molecular chaperones control these processes.

## Introduction

The microtubule-associated protein tau accumulates in a number of neurodegenerative diseases termed tauopathies, including Alzheimer's disease (AD), progressive supranuclear palsy (PSP), frontotemporal dementia and parkinsonism linked to chromosome 17 (FTDP-17), and several others. Many of these diseases are caused by missense mutations in the *MAPT* gene coding for tau, while several others are caused by environmental factors: chronic traumatic encephalopathy (CTE) is a sequelae of traumatic brain injury (TBI), postencephalitic parkinsonism (PEP) results from infection, while the cause of age-related AD is unknown. The sheer diversity of the factors that ultimately lead to tauopathy and neurodegeneration is quite remarkable and suggestive of a broad neurological reaction in response to a variety of insults. Because the disordered structure of tau lends itself to heavy post-translational modifications, signaling events caused by these environmental factors can have a multitude of effects on tau structural dynamics.

Tau aggregates into ß-sheet fibrils in tauopathies, leading to the formation of neurofibrillary tangles (NFTs) and subsequent cell death. Although precisely what triggers tau assembly into these ß-sheet structures in the brain is unclear, it is known that a number of post-translational modifications can regulate this process, including phosphorylation, acetylation, cleavage, ubiquitination, and misfolding. This mini-review will reveal what has been recently discovered about tau structure and how distinct cellular mechanisms such as molecular chaperones can control tau folding to promote or block its toxic assembly [for more comprehensive reviews on tau structure and pathology, we direct readers to Kolarova et al. (2012) and Wang and Mandelkow (2016)].

## Overview of tau structure

Tau is an intrinsically disordered protein with a strong propensity for self-aggregation into ß-sheet structures which compose the core of NFTs. Several factors can enhance the propensity of tau to aggregate, including mutations in the *MAPT* gene and post-translational modifications such as phosphorylation and acetylation (Goedert et al., 1988; Hutton et al., 1998; Spillantini et al., 1998; Von Bergen et al., 2001; Augustinack et al., 2002; Cohen et al., 2011; Mandelkow and Mandelkow, 2012; Cook et al., 2014; Min et al., 2015). Another post-translational modification to tau, ubiquitination, has been shown to be required for tau ß-sheet assembly *in vivo*; but when ubiquitination was blocked, soluble tau intermediates, typically termed oligomers, developed in the brain that were highly toxic (Dickey et al., 2006b). This idea of soluble tau oligomers being the major toxic species in the brain has gained recent support because of key studies that have emerged over the past decade (Santacruz et al., 2005; Oddo et al., 2006; Spires et al., 2006; O'leary et al., 2010). Using tools that have been developed to specifically investigate oligomeric tau species, it has been demonstrated that these structures were responsible for much of the neurotoxicity due to tau accumulation (Lasagna-Reeves et al., 2011; Blair et al., 2013). Moreover, several studies have shown that reducing soluble tau mitigates neuronal loss and functional deficits in tau transgenic mice, despite the lack of change in ß-sheet aggregates (Santacruz et al., 2005; Oddo et al., 2006; Spires et al., 2006; O'leary et al., 2010). Thus, it is clear that elucidating the processes governing tau oligomerization and aggregation is critical for not only understanding tau pathogenesis but also for developing tau-based therapeutics.

The structure of tau has been extensively analyzed in an effort to gain insight into the mechanisms of its aggregation and toxicity. Initial approaches using fluorescence resonance energy transfer (FRET) theorized a “paper clip” model of tau wherein the N and C termini transiently fold onto the microtubule-binding repeat domains as well as each other (Jeganathan et al., 2006). The tau protein contains either 3 or 4 microtubule-binding domain repeats (3R or 4R) which have been shown to be essential for both the ability of tau to bind to microtubules and its assembly into paired helical filaments (PHFs). Specifically, stretches of amino acid residues within these repeat domains, which include the hexapeptide motifs VQIIYK and VQIVYK, are capable of serving as seeds for aggregation (Von Bergen et al., 2001; Mukrasch et al., 2005). In fact, these motifs are uniquely critical for the intermolecular contact between tau molecules which gives rise to initial oligomer formation and eventual self-aggregation (Peterson et al., 2008). Moreover, the hexapeptide motifs have intramolecular contacts with proline-rich regions on tau, suggesting proline-directed phosphorylation may alter tau structure, affecting its aggregation propensity (Mukrasch et al., 2009). More recent work has corroborated this idea, demonstrating that intramolecular interactions in the repeat domains promote aggregation, whereas the more unstructured N terminus folds longer stretches which prevent aggregation (Wegmann et al., 2011). Disulfide cross-linking of tau can also play a substantial role in its aggregation propensity: 3R tau contains only one cysteine, oxidation of which permits cross-linking, oligomerization, and aggregation. Conversely, 4R tau contains two cysteines which readily form intramolecular contacts and can suppress cross-linking and aggregation (Schweers et al., 1995; Barghorn and Mandelkow, 2002). The consequences of these intra- and intermolecular tau interactions *in vivo* likely depend on a multitude of factors such as phosphorylation state, proteostatic burden, and binding to microtubules.

## Tau-microtubule interactions influence tau structure, assembly, and toxicity

It is likely not a coincidence that the regions within tau necessary for binding to microtubules are also important for ß-sheet assembly and aggregation. The first discovered function of tau was its ability to promote microtubule assembly (Weingarten et al., 1975) and subsequent studies have only reinforced these findings, showing that tau stabilizes microtubules with a high affinity through interactions within its microtubule-binding repeats (Goode et al., 1997; Sillen et al., 2007). The mechanism by which tau stabilizes microtubules was only very recently established using nuclear magnetic resonance (NMR) spectroscopy and mass spectrometry. Zweckstetter and colleagues revealed that tau binds to microtubules at the interface between tubulin heterodimers, using small groups of residues that have previously been shown to be critical for tau ß-sheet assembly and aggregation (Kadavath et al., 2015). The authors highlight the implication that competition may arise between the physiological interaction of tau with microtubules and tau misfolding and aggregation. Phosphorylation of tau can prevent its binding to microtubules, which could potentially give rise to a feed-forward loop under pathological conditions. In this scenario, hyperphosphorylation of tau, characterized by abnormal tau conformations, and self-assembly into PHFs, causes gain of toxicity as well as loss of function if it is unable to stabilize microtubules. Thus, loss of axonal stability coupled with tau self-assembly and aggregation contribute to neurodegeneration in tauopathies.

## Post-translational modifications influence tau structure

As discussed above, tau structure and conformation can be altered by several primary post-translational factors, but the best characterized of these are phosphorylation and proteolytic cleavage. Tau phosphorylation has a dramatic effect on the structure and function of tau, potentially obscuring the microtubule binding sites within the repeat domains. However, not all phospho-epitopes behave the same. For example, when tau is phosphorylated at the AT8 (S202/T205) and AT180 (T231) sites, it loses the ability to drive microtubule assembly, but can still associate with preformed microtubules (Amniai et al., 2009). In contrast, MARK-dependent phosphorylation of tau at S262 induces conformational changes around this phosphorylation site, altering tau structure and attenuating microtubule binding (Fischer et al., 2009). A recent investigation of tau phosphorylated at T231 revealed that this residue can selectively form a salt bridge with R230, which competes with the bridge necessary for tau-microtubule interaction (Schwalbe et al., 2015). Tau phosphorylation not only affects local conformations but has also been shown to weaken the transient long-range interactions common to intrinsically disordered proteins (Bibow et al., 2011; Sibille et al., 2012), potentially making tau more susceptible to aggregation. These studies and others have made it clear that phosphorylation of tau not only influences the aggregation potential of the protein, but can also have more immediate consequences on microtubule binding and folding.

Tau can also be modified by proteolytic cleavage events, altering its structure, functional capacity, and self-association. Tau can be cleaved by caspase-3, most notably at D421, or by the calcium-dependent protease calpain, which leads to a 17 kDa tau fragment (Hanger and Wray, 2010). Cleavage by either of these proteases produces truncated tau forms which are more neurotoxic than full-length tau (Chung et al., 2001; Park and Ferreira, 2005). Cleaved tau has been found in the brains of individuals with AD and other tauopathies, indicating it may play a role in disease pathogenesis (García-Sierra et al., 2008). Although most cleaved tau species that have been identified are C-terminal truncations, a recent report showed that an N-terminal truncation at Q124, identified from human brains via proteomics, actually enhanced microtubule stability (Derisbourg et al., 2015).

Evidence suggests that tau phosphorylation may also influence cleavage of tau; phosphorylation at S422 is commonly observed in tauopathy brains and has been shown to inhibit caspase cleavage of tau (Rissman et al., 2004; Guillozet-Bongaarts et al., 2006). Furthermore, tau phosphorylation and cleavage differentially affect the ability of tau to interact with microtubules (Drewes et al., 1997; Ding et al., 2006), suggesting tau may adopt an alternative structure that promotes aggregation following cleavage. It was also recently discovered that tau can be cleaved by asparagine endopeptidase, a lysosomal cysteine protease, which (1) abrogates the ability of tau to stabilize microtubules, (2) induces aggregation, and (3) enhances neurodegeneration (Zhang et al., 2014). Collectively, these studies suggest that post-translational modifications such as phosphorylation and cleavage can affect the ability of tau to interact with microtubules and make it more susceptible to self-assembly and aggregation. Further work may begin to develop a clearer picture of how the interplay between these modifications influences the propensity of tau to form ß-sheet structures and eventual irreversible aggregates. Other factors such as molecular chaperones also play an important role in determining tau fate both in concert with and independent of post-translational modifications.

## Chaperones control tau structure and assembly

Molecular chaperones such as Hsp70 and Hsp90 have been long recognized as vital mediators of protein folding and structure (Young et al., 2004). These proteins help maintain proteostasis by attempting to refold misfolded proteins or shuttling them to the proteasome if refolding is unsuccessful. A seminal paper by Hu and colleagues showed that Hsp70 and Hsp90 can each directly interact with tau, promoting tau-microtubule binding while decreasing tau phosphorylation and aggregation (Dou et al., 2003). Inhibition of Hsp70 or Hsp90 with siRNA or small molecules reduces intracellular tau levels in cells and the brain (Dickey et al., 2005, 2006a, 2007; Jinwal et al., 2009). The heat shock response typically produced by this inhibition increases levels of Hsp72, an inducible Hsp70 isoform, leading to enhanced tau turnover (Dickey et al., 2006a). In fact, it is known that Hsp72 and Hsc70, the predominant, constitutively active isoform of the Hsp70 family, have opposing effects on tau clearance, with Hsc70 preserving tau in the cell and Hsp72 promoting tau degradation (Jinwal et al., 2013).

Tau phosphorylation seems to be an important determinant of chaperone activity. The carboxyl terminus of the Hsc70-interacting protein (CHIP) is a major co-chaperone of Hsp90 and Hsc70. Tau phosphorylation is required for the Hsc70-CHIP complex to shuttle tau to the proteasome for ubiquitination, an event that mitigates cell death induced by tau hyperphosphorylation (Shimura et al., 2004). Similarly, the Hsp90-CHIP complex selectively degrades phosphorylated tau species (Dickey et al., 2007). However, these processes appear to be phospho-epitope specific; although tau phosphorylated at S202/T205 and S396/S404 could be degraded by Hsp induction, tau phosphorylated at S262/S356 was not affected (Dickey et al., 2006a). Interestingly, deletion of CHIP promotes accumulation of phospho-tau without promoting its aggregation (Dickey et al., 2006b). Thus, distinct chaperones clearly recognize structural features of tau to govern the degradation and assembly of tau. This point is illustrated by data demonstrating that Hsp70 and Hsp90 actually compete for binding to shared residues on tau, leading to differential effects on tau clearance; Hsp70 stabilizes tau levels while Hsp90 facilitates tau removal (Thompson et al., 2012). Hsp70 and Hsp90 also have opposing effects on tau assembly and structure *in vitro*. While Hsp70 prevents tau aggregation (Patterson et al., 2011), Hsp90 stimulates it (Blair et al., 2013). Because the dynamic chaperone landscape changes with age and especially AD (Yoo et al., 2001; Jinwal et al., 2010; Blair et al., 2013), it is likely that dysfunction in the ability of Hsp70 or Hsp90 to regulate tau contributes to its misfolding and aggregation.

Although Hsp90 is one of the more well-characterized chaperones, it was only recently discovered how it interfaces with a substrate (Karagoz et al., 2014). In fact, tau was the model substrate used to determine this. Through this work, it was found that Hsp90 interacts with a long stretch of the tau microtubule binding repeats, essentially stretching tau out along the N-terminal and middle domains of Hsp90 (Karagoz et al., 2014). Although tau is intrinsically disordered, it adopts conformations that facilitate its pathogenicity, a property that is referred to as “meta-stability” and Hsp90 may constrain this flexibility to some extent. But in doing so, Hsp90 can facilitate tau ß-sheet assembly. Of course Hsp90 does not work alone within the cell. The dynamic network of other major chaperones and co-chaperones can influence several aspects of tau biology. For example, the Hsp90 co-chaperones FKBP51 and FKBP52 have opposing effects on tau assembly, oligomerization, and aggregation (Blair et al., 2013; Giustiniani et al., 2015). Thus, a number of cellular factors coordinate to regulate tau structure and proteostasis; an imbalance in these factors, as occurs with aging, could shift regulation of tau to a more pathogenic state.

## Conclusions

To describe proteins like tau as intrinsically disordered may be somewhat misleading. Perhaps proteins with unstructured domains should rather be considered dynamically flexible, providing a way for the cellular proteome to adapt to distinct environments and needs. These metamorphic proteins come with a cost however, in that some structures can promote proteotoxicity. The factors that promote these toxic structures are critical for our understanding of tauopathies and could each be viable therapeutic targets. Moreover, if these transient structures can be identified, it could usher in a new era of structure-based drug discovery for tauopathies and other diseases associated with metamorphic proteins. With the advent of improved methods of 3D electron microscopy and high resolution mass spectrometry, examining these large, aggregate structures is now feasible and will certainly yield key insights for therapeutic development in these disorders.

## Author contributions

JS and CD each contributed to the writing of the manuscript.

## Funding

This work was supported by funding from the National Institute of Neurological Disorders and Stroke (NS073899).

### Conflict of interest statement

The authors declare that the research was conducted in the absence of any commercial or financial relationships that could be construed as a potential conflict of interest.
